# A microscopy study of hyphal growth of *Penicillium rubens* on gypsum under dynamic humidity conditions

**DOI:** 10.1111/1751-7915.12357

**Published:** 2016-03-21

**Authors:** Karel A. van Laarhoven, Hendrik P. Huinink, Olaf C. G. Adan

**Affiliations:** ^1^ Department of Applied PhysicsEindhoven University of TechnologyEindhoventhe Netherlands

## Abstract

To remediate indoor fungal growth, understanding the moisture relations of common indoor fungi is crucial. Indoor moisture conditions are commonly quantified by the relative humidity (*RH*). *RH* is a major determinant of the availability of water in porous indoor surfaces that fungi grow on. The influence of steady‐state *RH* on growth is well understood. Typically, however, the indoor *RH* constantly changes so that fungi have to endure frequent periods of alternating low and high *RH*. Knowledge of how common indoor fungi survive and are affected by the low‐*RH* periods is limited. In particular, the specific effects of a drop in *RH* on the growth of the mycelium remain unclear. In this work, video microscopy was used to monitor hyphal growth of *Penicillium rubens* on gypsum substrates under controlled dynamic humidity conditions. The effect of a single period of low *RH* (*RH* = 50–90%) interrupting favourable conditions (*RH* = 97%) was tested. It was found that hyphal tips ceased to extend when exposed to any tested decrease in *RH*. However, new hyphal growth always emerges, seemingly from the old mycelium, suggesting that this indoor fungus does not rely only on conidia to survive the humidity patterns considered. These findings are a fundamental step in unravelling the effect of *RH* on indoor fungal growth.

## Introduction

Indoor moulds may excrete metabolites or fungal particles that induce allergic reactions or asthma in some subpopulations (Miller, 1992; Flannigan, 2001; Green *et al*., 2011). Furthermore, mould discolours indoor surfaces. To avoid such medical or aesthetical problems, strategies for the remediation of indoor fungal growth are required. A detailed knowledge of the conditions leading to mould colonization of indoor surfaces forms the basis for such strategies.

Fungal growth is influenced by many abiotic factors, such as moisture, temperature, nutrient availability, oxygen, chaotropicity (Cray *et al*., 2013) and pH. Of these factors, temperature and especially moisture are considered the most important factors influencing indoor fungal growth (Grant *et al*., 1989; Adan *et al*., 2011). Most indoor surfaces consist of porous materials, which can absorb and store the water required for fungal growth. While indoor water can originate from leakage or flooding, the adsorption of water vapour from air with sufficiently high relative humidity (*RH*) onto a porous material can result in fungal colonization of surfaces (Coppock and Cookson, 1951; Clarke *et al*., 1999; Bekker *et al*., 2015). Several studies on the effects of moisture on indoor fungal growth have, therefore, focused on the dependence of growth on porous materials on the *RH* (e.g. Grant *et al*., 1989; Pasanen *et al*., 1992b; Adan, 1994; Chang *et al*., 1995; Viitanen and Bjurman, 1995; Chang and Foarde, 1996; Clarke *et al*., 1999; Viitanen *et al*., 2010; Johansson *et al*., 2013; Bekker, 2014; Bekker *et al*., 2015; van Laarhoven *et al*., 2015).

The minimal moisture requirements of a fungus are generally expressed in terms of the water activity (*a*_*w*_) of its environment (Scott, 1957). Fungal protoplasm has its own *a*_*w*_, and water transport occurs from high to low *a*_*w*_. Water uptake, which is crucial for cell processes and structure (Griffin, 1981; Magan, 2007), can therefore occur only when the internal *a*_*w*_ is below the *a*_*w*_ of the environment. *RH* and *a*_*w*_ are related: both are expressions of water's chemical potential, in the vapour and condensed phase respectively (Atkins and de Paula, 2006). Therefore, when an indoor substrate and ambient *RH* are in equilibrium, the *RH* defines the substrate *a*_*w*_ via *a*_*w*_ = *RH*/100%. The influence of a decreasing steady‐state *a*_*w*_ or *RH* on growth has been extensively investigated on agar media (e.g. Ayerst, 1969; Magan and Lacey, 1984; Judet *et al*., 2008; Nanguy *et al*., 2010; Ponizovskaya *et al*., 2011) and on various building materials (e.g. Grant *et al*., 1989; Pasanen *et al*., 1992b; Nielsen *et al*., 2004; Viitanen *et al*., 2010). While the exact relationship depends on the fungal species and substrate material, a general trend of slower germination and hyphal growth with lowered *a*_*w*_ or *RH* has been found, down to a system‐specific critical value, below which no growth occurs (Stevenson *et al*., 2015).

Typically, the indoor *RH* is not constant, but rather fluctuates due to indoor temperature gradients and due to household activities such as cooking or bathing. While the indoor humidity is below the *RH* threshold for mould growth on average, it has been shown that these transient periods of high *RH* are sufficient for sustaining mould growth on indoor surfaces (Pasanen *et al*., 1992a; Adan, 1994; Viitanen *et al*., 2010; Johansson *et al*., 2013; Johansson, 2014). A full picture of indoor fungal growth, therefore, needs to include detailed knowledge of how the fungi survive and respond to the recurring periods of low *RH* that interrupt the humidity conditions suitable for growth.

Several authors have studied the effect of transient humidity on fungal growth on various building materials, such as gypsum (Adan, 1994; Bekker, 2014), wood (Viitanen, 1997; Johansson *et al*., 2013) or concrete (Viitanen and Ojanen, 2007). These workers each performed growth experiments in which fungi on porous substrates were exposed to a cyclic regime of alternating periods of high and low *RH*. Because the tested materials, moisture regimes and fungal species varied per study, direct comparison of these studies is difficult (Vereecken and Roels, 2012; Dedesko and Siegel, 2015). In general, however, each of these researchers found that exposure to a cyclic *RH* lowers the rate of fungal proliferation during the periods of high *RH* as compared with proliferation during the same *RH* at steady‐state conditions.

Bekker (2014) focused on the effects of a single period of low *RH* on the growth of *Penicillium rubens* on gypsum substrates. She found that low *RH* periods with different characteristics, such as duration and moment of application, influence growth differently. Moreover, Bekker's results indicate that mycelium remains viable during short periods of low *RH* (< 48 h), whereas regrowth on substrates exposed to longer periods of low *RH* seemed to originate only from conidia that germinated post‐desiccation. A limited number of studies on agar have reported similar phenomena (Diem, 1971; Luard, 1982; Park, 1982; Browning *et al*., 2008; Bekker, 2014). For growth on porous materials, however, a sound interpretation is hindered by the lack of direct microscopic data. The studies so far have used macroscopic methods to quantify growth, that is, methods based on measurement of macroscopic properties of fungal colonies as a whole. An important method used in many previous studies is the assessment of surface coverage with either stereoscopy (Adan, 1994; Viitanen *et al*., 2010; Johansson, 2014) or digital images (Nielsen *et al*., 2004; Bekker, 2014). As such, the effects of transient humidity conditions on the individual stages of fungal growth, that is, germination, hyphal growth or sporulation, remain unclear.

The aim of this work was to explore how hyphal extension on porous materials is influenced by a single period of low *RH*. Video microscopy experiments (van Laarhoven *et al*., 2015) are reported in which hyphal growth of the indoor mould *P. rubens* (Andersen *et al*., 2011; Samson, 2011) on gypsum, a common porous building material, was recorded during and after its exposure to low *RH*. Knowledge of the hyphal response to *RH* changes will produce a more complete understanding of indoor fungal growth and will aid the interpretation of macroscopic studies on indoor fungal moisture relations. Ultimately, this may lay the foundations for new control strategies for indoor fungal growth.

## Method

### Gypsum substrates

The substrates for the growth experiments were made by mixing gypsum ((Ca_2_SO_4_)_2_·H_2_O, Sigma Aldrich, St. Louis, Missouri, United States) with an aqueous solution of Czapek Dox Borth (Oxoid, 8.76 g l^−1^) and the trace metals ZnSO_4_·7H_2_O (2.5 × 10^−3^ g l^−1^) and CuSO_4_·5H_2_O (1.25 × 10^−3^ g l^−1^). The solution was first autoclaved, then mixed with the calcium sulphate hemihydrate at a mass ratio of 2:3, and finally cast into 3 mm thick moulds. The samples were cured and dried for 48 h at room temperature in a Bio Safety Cabinet (BSC) (CleanAir, Class II – EF/B) to remove excess water.

The surface of each sample was coloured with a thin layer of Fe_3_O_4_ (Metzger Black), to provide sufficient contrast for microscopy. The layer was applied by pipetting 5 μl of a Fe_3_O_4_ suspension in water (33.3 g l^−1^) on to the substrate. The samples were then again stored in the BSC until the suspension water had evaporated. The resulting Fe_3_O_4_ layer typically had a thickness of about 20 μm, as determined with a profilometer (confocal interferometer, Smart; Sensofar, Barcelona, Spain). Inspection with cryo‐SEM showed that the layer was porous with typical pore sizes in the order of 0.1–1 μm (van Laarhoven *et al*., 2015).

### Fungal strain, conidial suspension and inoculation

*Penicillium rubens* (strain CBS 401.92; CBS Fungal Biodiversity Centre, Utrecht, The Netherlands) was used as the test organism in this study. This strain was formerly known as *Penicillium chrysogenum*, but was reclassified in 2011 (Houbraken *et al*., 2011).

Stock conidial suspension was created as follows. Conidia were collected by wetting 1 week old *P. rubens* cultures on MEA with autoclaved, demineralized water with 0.05 vol% Tween80 and subsequently scraping the surface of the colonies. The resulting conidial suspension was filtered with sterile glass wool to remove mycelium fragments. The suspension was then pelleted three times by centrifugation, each time followed by washing with autoclaved demineralized water with 0.05 vol% Tween80. Finally, 30 vol% of sterile glycerol was added to the suspension. The final concentration of the stock was counted using a hemocytometer, being 1.6 × 10^8^ ml^−1^. The stock was subsequently stored at −30°C.

Gypsum substrates were inoculated with conidial suspension of *P. rubens* as follows. An amount of stock was unfrozen and diluted to a concentration of 10^6^ ml^−1^ before use. The area on the substrate coloured with Fe_3_O_4_ was then inoculated by pipetting 5 μl of spore suspension onto it. In this way, approximately 5000 spores were evenly distributed over an area of approximately 10 mm^2^. Inoculated samples were then dried in the BSC for about 20 min to evaporate the suspension water. Consequently, the occurrence of a non‐equilibrium water distribution near the inoculum during experiments was prevented. An analytic balance (Mettler Toledo, Columbus, Ohio, United States) was used to confirm that at least 95% of the suspension water had evaporated before incubation of the samples.

### Incubation and *RH* control

**Figure 1:**
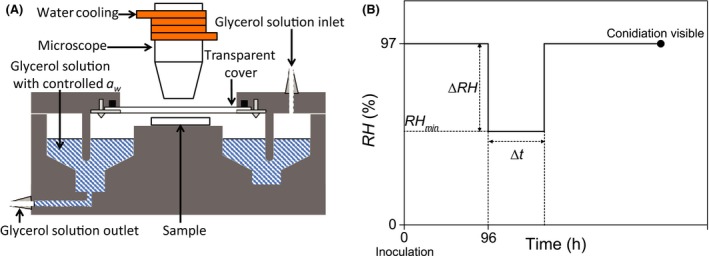
Schematic representation of the setup for growth experiments. (A) An inoculated sample is stored in the incubation chamber above a glycerol solution that controls the chamber's *RH*. Growth on the substrate is recorded with video microscopy through the transparent lid of the chamber. The *RH* in the container is controlled by replacing one glycerol solution with another with pumps connected to the inlet and outlet. The setup is placed in a constant temperature room; the temperature in the incubation chamber is 23.3 ± 0.1°C. The temperature difference between sample and solution is at most 0.05°C. (B) Schematic representation of the sequence of humidity steps during experiments. Directly after inoculation at *t* = 0, samples are stored at *RH* = 97% for 96 h. Then, samples are exposed to a period of lower *RH* with value *RH*_min_ with duration Δt. The *RH* returns to 97% after the period of low *RH*, and the experiment continues until conidiation is observed on the sample.

The inoculated samples were stored in small incubation chambers (Fig. 1). The *RH* inside these chambers was controlled with an aqueous glycerol solution of defined concentration and *a*_*w*_ on the bottom of the container, below the samples (Forney and Brandl, 1992). An *a*_*w*_‐meter (Labtouch‐aw Basic, Novasina, Lachen, Switzerland) was used to verify the *a*_*w*_ of the solutions with an accuracy of ± 0.01 (equivalent to ± 1% *RH* in the incubation chambers). Syringe pumps (NE‐1600; New Era, Farmingdale, New York, United States of America) were used to replace one glycerol solution with another, thereby dynamically controlling the *RH*. The containers were airtight to prevent the development of inhomogeneous *RH* profiles and to prevent evaporation‐induced changes in the concentration and *a*_*w*_ of the glycerol solutions. Moreover, verification of the *a*_*w*_ of solutions before and after experiments confirmed that their *a*_*w*_ did not change noticeably during the experiments. The setups were kept in a constant temperature room and cameras were water cooled so that the setups had a uniform, constant temperature (23.3 ± 0.1°C), as verified with 4 thermocouples (NI USB‐9213; National Instruments, Austin, Texas, United States) distributed across the incubation chamber. This again ensured that homogeneous *RH* profiles developed throughout the whole container after each change of glycerol solution.

**Figure 2:**
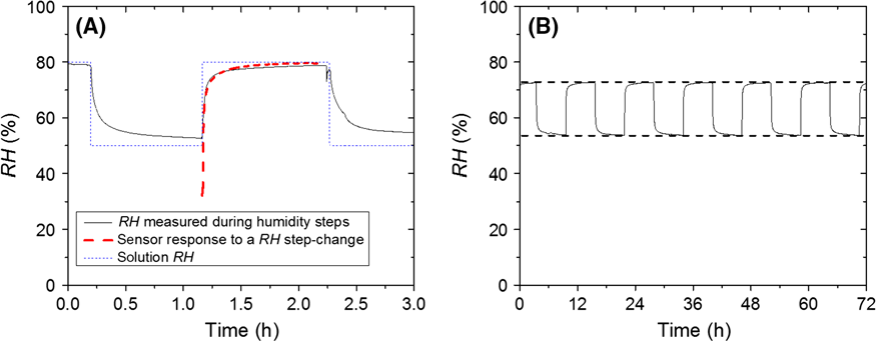
The *RH* on the position of the sample, measured as a function of time with a humidity sensor (continuous line). (A) Glycerol solutions of alternatingly *a*_*w*_ = 0.8 and *a*_*w*_ = 0.5 are brought into the chamber, imposing a switching equilibrium *RH* (dotted line). The dashed line is the sensor response when transferred from dry air into a container pre‐equilibrated at *RH* = 80%. (B) Glycerol solutions of alternatingly *a*_*w*_ = 0.76 and *a*_*w*_ = 0.53 are brought into the chamber, replacing each other every 6 h.

Equilibration of the *RH* throughout the incubation chamber after each change of glycerol solutions was driven by vapour diffusion; no additional stirring was applied. This was done to keep hyphae on the surface unperturbed by moving air, making it possible to follow hyphal extension with video microscopy. A control measurement during a change of solutions from *RH* = 97% to *RH* = 97% indicated that hyphae indeed remained undisturbed by the mechanical process of changing solutions. To confirm that diffusion facilitated sufficiently fast equilibration of the chamber, the *RH* at the location of the sample was measured as a function of time with a *RH* sensor (SHT71; Sensirion, Staefa, Switzerland) after switching between glycerol solutions (Fig. 2A). Equilibrium is reached within approximately 15 min and possibly faster, since the measurement is limited by the response time of the sensor (bold dashed line). The *RH* was also measured during cyclic replacement of two solutions for several periods to confirm that the same *RH* value was reached every time (Fig. 2B).

### Quantification of fungal growth on the gypsum substrates

During the experiments, samples were observed through the transparent lid of the container with a USB‐microscope (Dino‐Lite 7013MZT (AnMo Electronics Corporation, New Taipei City, Taiwan), numeric aperture 0.22, optical resolution ~1.5 μm). A magnification of 470× was used, corresponding to a field of view (FOV) of 0.84 mm × 0.63 mm and a pixel size of 0.6 μm × 0.6 μm. The contrast provided by the Fe_3_O_4_ layer on the substrate allowed the observation of hyphal growth on the samples. It is stressed that germination and germ‐tube formation could not be resolved: only developed hyphae growing above but parallel to the surface can be followed (van Laarhoven *et al*., 2015) The thickness of the microscope working plane was ~75 μm, as determined with a micromanipulator (Leica micromanipulator, Leica Leitz, Wetzlar, Germany). Growth was monitored at 1 h intervals with time‐lapse recording.

**Figure 3:**
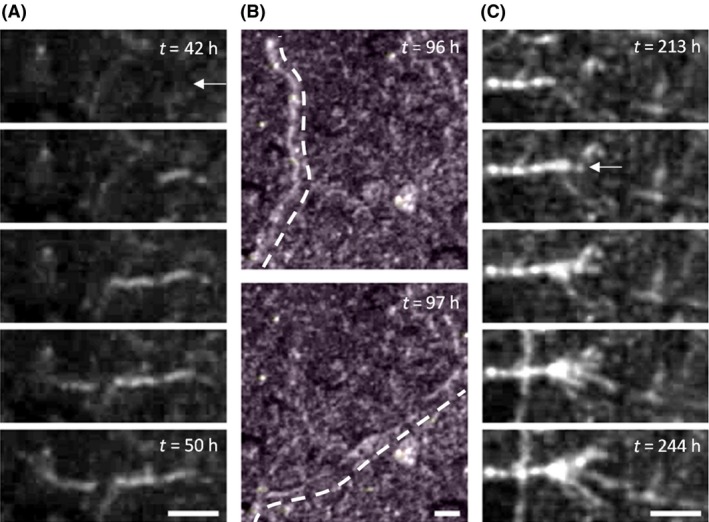
Cropped frames of a typical movie produced with the video setup. The scale bars represent 25 μm. (A) A hypha growing on a gypsum sample exposed to *RH* = 97%, at 2 h intervals. The arrow marks the moment right before the hypha becomes visible. (B) A hypha severely displaces right after a change in *RH* from 97% to 90%. (C) Formation of a *P. rubens* fruiting body at 8 h intervals. The arrow marks the moment of sporulation, defined as the last frame in which no conidiophores can be recognized.

**Figure 4:**
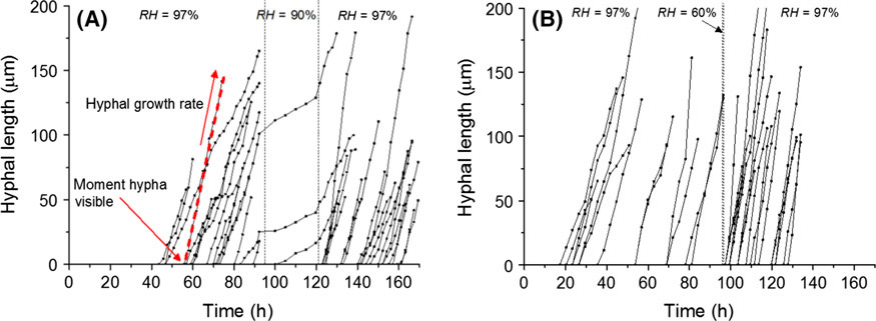
Hyphal length as a function of time for hyphae growing on gypsum samples while exposed to a single period of lowered *RH* with Δ*t* = 24 h, *RH*_min_ = 90% (a) and Δ*t* = 1 h, *RH*_min_ = 60% (B). Per figure, the data were obtained from tracing hyphae in two movies of growth on samples exposed to the low *RH* period. The dotted lines indicate the period of exposure to *RH*_min_. The growth rate and moment of appearance of each individual hypha are determined with a linear fit, as illustrated with the dashed line in (A). *t* = 0 corresponds to the moment of inoculation.

The recorded movies were post‐processed to quantify the observed hyphal growth. A custom MATLAB script was used to trace the position of individual hyphal tips from series of frames like those shown in Fig. 3A. Hyphal length as a function of time, starting from the first sight of growth, could be determined accordingly. An example of such data is displayed in Fig. 4, which shows the length of 37 individual hyphae that were traced from two movies of a full measurement, plotted as a function of time with *t* = 0 the moment of inoculation. The dotted lines mark the period of low *RH*.

Based on the movies, three aspects of growth were quantified. First, the growth rate of each individual hypha was determined on the basis of a linear fit, as illustrated in Fig. 4A. Second, fitting of the hyphal length also allowed the identification of the first moment in time each hypha becomes visible in the FOV (shown by the arrow in Fig. 3A), that is, the point in time where the observed hyphal length in Fig. 4 is equal to 0. Third, for each movie, the moment of conidiation was defined as the timestamp of the last frame in which no conidiophores could be recognized in the FOV (shown by the arrow in Fig. 3C).

### Growth experiments

The *RH* exposure of samples during growth experiments is illustrated in Fig. 1B. All samples were initially incubated at *RH* = 97%. After 96 h, samples were exposed to a lower *RH* of certain value *RH*_min_ for a period with duration Δ*t* (Fig. 1B). The *RH* was switched back to 97% at the end of the period of low *RH*.

To investigate the influence of *RH*_min_, a series of experiments was performed in which *RH*_min_ was set at a fixed and constant value of 50%, 60%, 70%, 80% or 90%.

To investigate the influence of Δ*t*, the measurement series described above was carried out twice, with Δ*t* set to 24 h and 1 h respectively.

As a reference, growth at a steady‐state *RH* = 97%, that is, uninterrupted by a period of low *RH*, was measured as well.

Unless otherwise noted, the period of low *RH* was initiated 96 h after incubation. However, during the 96 h of fungal growth at *RH* = 97% prior to the application of a low *RH* period, conidiophores bearing new conidia might already form in the more mature mycelium. To exclude the possible role of these newly formed conidia, additional measurements were performed in which a period of *RH*_min_ = 50% or 90% and Δ*t* = 24 h was applied 48 h after inoculation.

After the period of low *RH*, recording of growth on the samples continued until conidiation was visible in the FOV. After that, a measurement was terminated because growth at that point was typically so advanced that hyphae overlapped extensively and could not be followed. Measurements were performed in triplicate for every combination of *RH*_min_ and Δ*t*.

### Statistical analysis

The general trends in the growth rates as a function of *RH*_min_ were investigated for experiments with both Δ*t* = 24 h and Δ*t* = 1 h. Since only trends were tested and no regression was attempted, *RH*_min_ was treated as a categorical variable. Sample sizes were generally unequal and homogeneity of variances was not assumed, so a Welch's one‐way ANOVA was used, followed by a Games‐Howell post hoc analysis. All statistical analysis was performed in Microsoft Excel 2010. An alpha level of 0.05 was used for all statistical tests.

Linear fits of the length of hyphae as a function of time were performed with MATLAB.

## Results

### Main observations before, during and after application of a period of low *RH*

**Figure 5:**
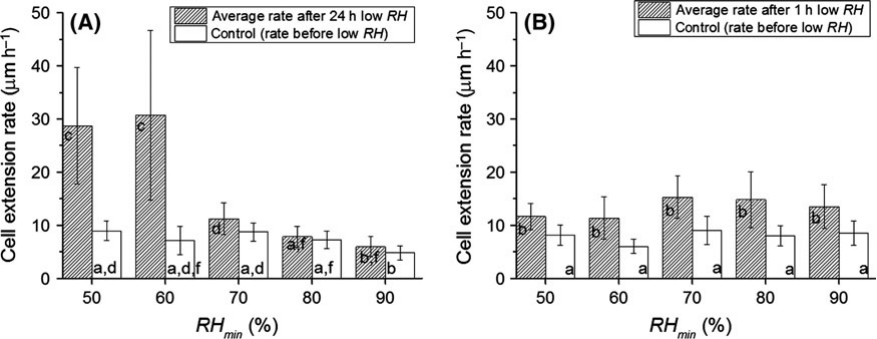
Average growth rates of hyphae growing on a gypsum substrate, before and after exposure to a period of *RH*_min_ with duration Δ*t* of 24 h (A) or 1 h (B). *RH* = 97% before and after the period of low *RH*. Error bars represent the standard deviation in the individual results. Welch's one‐way ANOVA shows that the averages are significantly heterogeneous (*F*_9,55_ = 30, *P* = 2.7e‐18) for (A), (*F*_9,43_ = 14, *P* = 3.69e‐18) for (B). Averages with a different label (a, b,…) are statistically different (Games‐Howell post hoc tests, *P* = 0.05).

Figure 3 displays several time labelled images of growth on the samples during a typical experiment (Δ*t* = 24 h, *RH*_min_ = 90%). The images are cropped frames of the recorded movies. A typical example of an extending hypha as observed in the movies is shown in Fig. 3A. Growth rates measured during the initial 96 h of steady‐state *RH* = 97% (Fig. 1) served as a reference point for each experiment, justified on the basis of comparison with the steady‐state experiments. The average of these initial growth rates is included per experiment in Fig. 5. Welch's one‐way ANOVA shows that the average initial growth rates are statistically heterogeneous (*F*_10,49_ = 5, *P* = 2.9e‐5). However, further analysis (Games‐Howell post hoc tests, *P* = 0.05) showed that the average initial growth rate of any experiment did not differ significantly from the average growth rate during steady‐state growth at *RH* = 97%, which was 8 ± 3 μm h^−1^.

The movies for which Δ*t* = 24 h show that within an hour after initiation of the low *RH* period (Fig. 1), hypha cease growing and are suddenly displaced, as shown in Fig. 3B. Also, no new growing hyphae appeared during the low *RH* period. The sole exception to this occurred for *RH*_min_ = 90% and Δ*t* = 24 h. In that case, three hyphae continued to grow during the period of low *RH* at a low growth rate (0.7 ± 0.3 μm h^−1^), as shown in Fig. 4A.

In the case of Δ*t* = 1 h, the period of low *RH* was too short to register whether hyphae were still growing during the period of low *RH*. However, hyphae growing before the low *RH* period could be seen to displace at the initiation of low *RH* and did not continue growing from that point onwards (See Fig. 4B). The only exceptions occurred in the cases of a *RH*_min_ of 90% or 80%, where, respectively, five and two hyphae were observed to grow prior to and after the low *RH* period.

For every combination of *RH*_min_ and Δ*t* during experiments, hyphal growth eventually reinitiated on all samples after the *RH* was brought back to 97% (Fig. 1). It is stressed that hyphal tips that ceased growth during the low *RH* period did not resume growth: all hyphae that were seen to grow in the second period of *RH* = 97% (apart from the exceptions mentioned above) appeared as newly detected hyphae such as illustrated in Fig. 3A. The regrowth during the second period of *RH* = 97% was influenced by the characteristics of the low *RH* period, as will be discussed in the following sections.

### Influence of a period of lower *RH* on subsequent hyphal growth rates

Figure 5 shows the average hyphal growth rates before and after the low *RH* period as a function of *RH*_min_. For Δ*t* = 24 h (Fig. 5A), the growth rate before and after the low *RH* period did not differ significantly for a *RH*_min_ of 70%, 80% or 90%. The average growth rates after periods of *RH*_min_ = 50% and *RH*_min_ = 60%, however, are substantially higher than the rates before the low *RH* period (~8 ± 3 μm h^−1^ before and ~30 ± 15 μm h^−1^ after).

For Δ*t* = 1 h (Fig. 5B), the post‐*RH*_min_ growth rates are significantly higher than pre‐*RH*_min_ growth rates for each *RH*_min_. The response to a *RH*_min_ of 50% or 60%, however, does not differ significantly from the other responses, as in the case for Δ*t* = 24 h.

### Influence of a period of lower *RH* on the moment of initiation of subsequent growth

**Figure 6:**
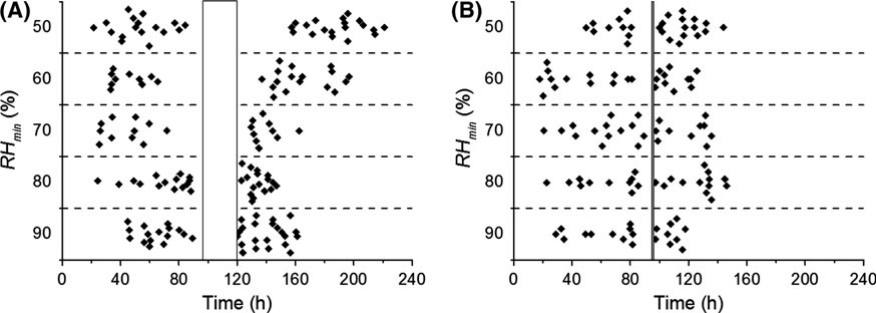
The initial times at which individual hyphae became visible in the FOV of the microscope, grouped per *RH*_min_. (A) The results from the experiments in which Δ*t* = 24 h. (B) The results from the experiments in which Δ*t* = 1 h. The vertical bars indicate the period of low *RH*.

Figure 6A shows the moments in time at which individual hyphae became visible in the FOV, grouped per *RH*_min_. For Δ*t* = 24 h (Fig. 6A) and a *RH*_min_ of 80% or 90%, the first new hyphae become visible within an hour after the end of the low *RH* period and from then, gradually, more hyphae keep appearing. The appearance of the first new hyphae following a *RH*_min_ of 70%, 60% or 50%, on the other hand, is delayed by 10, 20 or 40 h respectively.

For Δ*t* = 1 h (Fig. 6B), new growing hyphae become visible within 1 h after the end of the low *RH* period for all *RH*_min_ and from then, gradually, more hyphae keep appearing (also see Fig. 4B).

### Influence of a period of lower *RH* on subsequent conidiation

**Figure 7:**
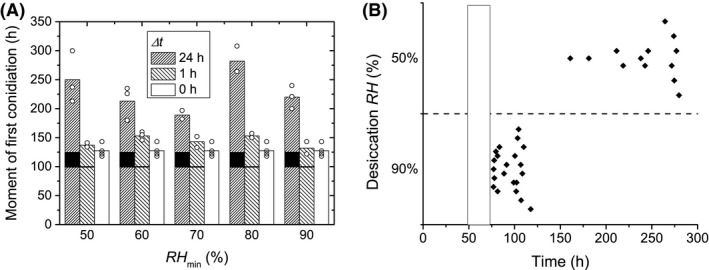
(A) The initial moments in time when conidiation became visible in the microscope's FOV (Fig. 3C) on samples that were exposed to a period of *RH*_min_ for Δ*t*. The first observation of conidiation was measured with respect to *t* = 0, the time of inoculation. Circles mark the times at which the first conidiophores were observed in individual movies, bars mark the average value. The cases Δ*t* = 0 h and Δ*t* = 1 h were not significantly different, but the case of Δ*t* = 24 h was statistically different from both (Welch's one‐way ANOVA,* F*_2,10_ = 32, *P* = 3.7e‐5 and Games‐Howell post hoc tests, *P* = 0.05). The amount of data points per *RH*_min_ was too small for further statistical analysis. The black area on a bar indicates the period of low *RH*. (B) The times at which individual hyphae became visible when a 24 h period of lower *RH* was applied 48 h after inoculation, grouped per *RH*_min_. The vertical bar indicates the period of low *RH*.

The times when conidiation was observed in each movie were collected and shown in Fig. 7A. The data are grouped by *RH*_min_ and Δ*t*; the moment of conidiation for growth at steady‐state *RH* = 97% is included as well. Comparison of the average conidiation times for Δ*t* = 24 h, Δ*t* = 1 h and Δ*t* = 0 h (steady‐state) shows no significant difference between the cases of Δ*t* = 1 h and Δ*t* = 0 h, but also shows that conidiation is significantly delayed in the case of Δ*t* = 24 h compared with both other cases (Welch's one‐way ANOVA, *F*_2,10_ = 32, *P* = 3.7e‐5 and Games‐Howell post hoc tests, *P* = 0.05).

Further statistical analysis to find a trend in conidiation times with *RH*_min_ was not appropriate due to the small amount of data points per *RH*_min_, which was limited by the number of movies. It is important to note, however, that the delay in conidiation for all cases of Δ*t* = 24 h was more than 48 h, that is, twice longer than Δ*t*.

### Regrowth after a low *RH* period applied 48 h after inoculation

The average growth rates of hyphae growing during the second period of *RH* = 97% after a period of Δ*t* = 24 h, applied after 48 h since inoculation, were 8 ± 3 μm h^−1^ and 22 ± 4 μm h^−1^ for *RH*_min_ = 90% and *RH*_min_ = 50% respectively. Furthermore, new growing hyphae appeared immediately after the period of *RH*_min_ = 90%, but was markedly delayed after the period of *RH*_min_ = 50%, as is shown in Fig. 7B.

## Discussion

### Hyphal response to a sudden decrease in *RH*

The main consequence of hyperosmotic shock to a fungus is an outflow of water that results in loss of turgor (Deacon, 2006). On agar, immediate loss of turgor in filamentous fungi has been shown to cause the hyphae to loose rigidity, shrink and curl up (Park, 1982; Deacon, 2006; Lew, 2011). This may explain the observed shifting of hyphae right after the initiation of the period of low *RH*, which essentially constitutes a hyperosmotic shock.

The steady‐state *RH* below which no growth occurs was previously determined to be ~86% for specifically *P. rubens* on gypsum (Adan, 1994; van Laarhoven *et al*., 2015). This means hyphal growth could be expected during a period with *RH*_min_ = 90% (Fig. 4A) but not during *RH*_min_ ≤ 80%. It is noted that the hyphal growth rates observed here during *RH*_min_ = 90% (0.7 ± 0.3 μm h^−1^) fall within the spread of hyphal growth rates previously observed for *P. rubens* on gypsum at steady‐state *RH* = 90% (at steady‐state *RH* = 90%, van Laarhoven *et al*., 2015). Still, even for *RH*_min_ = 90%, only 2 out of 9 followed hyphae continued growing past the initiation of the low *RH* period. It is likely that the other hyphae lost turgor and therefore ceased growing. On agar, Luard (1982) observed that *P. chrysogenum*, a fungus very closely related and similar to *P. rubens* (Houbraken *et al*., 2011), took 8 h to regain turgor and resume growth after a decrease in *a*_*w*_ from 0.99 to 0.95. After a decrease from 0.99 to 0.93 or lower, however, she observed no recovery of growth at all. This matches our observation that hyphae that cease growth do not recover during the 24 h period of *RH* = 90%.

### Resumption of hyphal growth following rewetting after a period of low *RH*

Our results indicate that for any considered combination of Δ*t* and *RH*_min_, the mycelium that developed prior to the period of low *RH* remains viable and produces new hyphae upon rewetting. We suggest this based on the following observations.

First (A), following the end of all low *RH* periods with Δ*t* = 1 h, several new growing hyphae appeared within an hour (Fig. 6B). The same occurred after periods of *RH*_min_ ≥ 80% and Δ*t* = 24 h (Fig. 6A). The fast appearance of these growing hyphae indicates that they originate from the previously developed mycelium.

Second (B), after periods of *RH*_min_ ≤ 60% with Δ*t* = 24 h, appearance of the first new hyphae was delayed for 20 h or more. However, regrowth occurred at a highly increased extension rate. The dramatically increased extension rate indicates a clear response to the low *RH* period. This suggests that this regrowth originates from structures that have retained some memory of the period of low *RH*, that is, the mycelium.

Based on the delayed conidiation after a period of low *RH* with Δ*t* = 24 h (Fig. 7A), one might alternatively conclude that all regrowth in this case stems from previously ungerminated conidia rather than the old mycelium: a completely new colony has to develop before conidiation can occur. Such a response to a period of low *RH* was, for instance, previously suggested by Bekker (2014). It is stressed, however, that conidiation is also delayed in the cases of Δ*t* = 24 h, *RH*_min_ = 80% or 90%, which are cases where hyphal growth resumes immediately upon rewetting after the period of low *RH*. Thus, a delay in conidiation should not necessarily be equated to the unviability of the old mycelium.

Still, it is stressed that, apart from regrowth from the old mycelium, it is possible that previously ungerminated conidia might, eventually, germinate and contribute to regrowth following a period of low *RH*. This cannot be excluded, as the currently used methods could not detect conidia or germ‐tube formation. Germination cannot, however, explain the immediate appearance of hyphae in case A. Further, it is unlikely that previously ungerminated conidia would respond to a period of low *RH* with an elevated growth rate like in case B, since their primary function is to withstand even more stressful moisture conditions (Griffin, 1994; Wyatt *et al*., 2013). Moreover, Bekker (2014) purposefully exposed ungerminated conidia of *P. rubens* on gypsum to similar moisture regimes and observed no differences in growth from those as compared with growth from conidia under a steady‐state *RH* = 97%.

Apart from previously ungerminated conidia, newly formed conidia might also contribute to regrowth eventually. In the case of a low *RH* period being applied after 96 h of growth at *RH* = 97%, new conidia might have already formed, as shown by an SEM study of Bekker *et al*. (2012) who observed that conidiation of *P. rubens* on gypsum at *RH* = 97% could occur as early as 92 h after inoculation. The results shown in Fig. 7B, however, indicate that hyphal growth responds similarly to a low *RH* period applied at 48 h after inoculation as to one applied after 96 h. This suggests that possible newly formed conidia are not crucial to this response, as the response also occurs in their absence.

Summarizing, we therefore conclude that, for all *RH* treatments considered, it is plausible that the previously developed mycelium produces new hyphae. It should be noted that hyphal tips that cease growth at the start of a low *RH* period did not resume growth afterwards. Therefore, it is likely that the observed new hyphal growth stems from branching from the old mycelium, forming new tips. Luard (1982) similarly observed regrowth of *P. chrysogenum* colonies on agar via branching, which occurred 250 μm behind the leading edge of the colony, 2 h after a hypoosmotic shock from *a*_*w*_ = 0.93 to *a*_*w*_ = 0.98. This conclusion does not match the findings of Park (1982), who exposed *P. chrysogenum* on agar to a more severe 168 h desiccation period at *RH* ~60%. He observed that 50 h after the desiccation, hyphal growth resumed from the centre of the colony, from which he concluded that the new growth stemmed from previously ungerminated conidia from the inoculum. The hyphal responses to desiccation reported here, however, cannot be explained with previously ungerminated conidia being the only source of regrowth after desiccation.

### Increased hyphal growth rates after a period of low *RH*

The mechanism that led to the enhanced growth rate of hyphae that grew after all periods of duration Δ*t* = 1 h and the periods of Δ*t* = 24 h with *RH*_min_ ≤ 60% is unclear and explanations are speculative. One possible explanation might be that the severe period of low *RH* destroy a large part of the previously developed mycelium. The contents of the destroyed mycelium might then provide additional resources for the few surviving hyphae.

Another explanation might be a modification of the substrate by the desiccation. When the porous gypsum is dried fast, it might result in transport of additional Czapek nutrients to the surface of the sample, as has previously been shown for the drying of aqueous solutions in porous media (e.g. Gupta, 2013). Additional steady‐state growth experiments on samples pre‐treated with a similar *RH* periods (data not shown), however, did not reveal a visible increase in growth rates.

Another possible explanation involves the fungus actively increasing its hyphal extension rate in response to the lower *RH*. It is known that fungi can direct their biomass increase by regulating the allocation of resources in a trade‐off between hyphal branching and hyphal extension (e.g. Trinci, 1974; Prosser and Tough, 1991; Heaton *et al*., 2012). Typically, hyphal extension rate is favoured over branching frequency in stressful or nutritionally sparse conditions so that the fungus' foraging capabilities are increased. Such a reaction might have been triggered by the lower *RH*. This could not be confirmed with the method used here, as the quality of the images was unsuitable for the collection of data on branching and total biomass production.

### Delay of conidiation after a long period of low *RH*

Compared with growth on samples that were exposed to steady‐state *RH* = 97%, conidiation was delayed by more than 48 h by a low *RH* period of Δ*t* = 24 h, whereas Δ*t* = 1 h did not delay conidiation significantly. To our knowledge, a common expectation is that water stress leads to earlier conidiation in fungi, although little literature is available on this subject (Abdel‐Hadi and Magan, 2009; Duran *et al*., 2010). Our observation that conidiation of *P. rubens* on gypsum is delayed by a 24 h exposure to even mildly lower *RH* therefore does not match with this expectation. More research is needed to explain this mismatch.

Insufficient data points were collected per *RH*_min_ to perform a statistical test for trends with *RH*_min_, but it is stressed that conidiation is also delayed in the cases of Δ*t* = 24 h, *RH*_min_ = 80% or 90%, which are cases where hyphal growth resumes immediately upon rewetting after the period of low *RH*. The knowledge that conidiation may respond differently to moisture history than the progression of mycelial growth is important for the interpretation of experiments in which the assessment of growth depends on the visibility of conidia (e.g. Nielsen *et al*., 2004; Bekker, 2014). Bekker, for instance, performed growth experiments with *P. rubens* on gypsum in which she assessed substrate discolouration after desiccation. From a delay in conidiation after a severe desiccation (48 h, *RH*_min_ = 15%), she concluded that regrowth originated only from previously ungerminated conidia. In contrast, the data on hyphal growth rates presented here indicate that part of the regrowth may stem from the previously established mycelium even while conidiation is delayed.

### Concluding remarks

In conclusion, we have identified several ways in which a period of low *RH* influences hyphal growth of *P. rubens* on gypsum for the first time. First, growing tips exposed to a desiccation of any considered duration or *RH* become unviable for further growth afterwards. Second, however, growth of new hyphae will eventually occur. Third, at least part of these new hyphae originate from the mycelium that developed prior to the low *RH* period, which indicates that parts of the mycelium other than the tips remain viable during all moisture regimes considered. Fourth, a period of low *RH* can change the hyphal growth rates afterwards. Finally, both the moment of production of new hyphae and that of new conidia can be delayed by a period of low *RH*, which indicates that current fungal growth can be dependent on past moisture conditions.

It is stressed that the evidence for regrowth from the old mycelium after a period of low *RH* is indirect, in the sense that it is inferred from the measured timescales that are involved with regrowth after the low *RH* period. The conclusions presented in this work could be strengthened by using stronger microscopy methods that are able to record branching of the mycelium directly, although this will not be trivial in the case of porous substrates.

A limitation of the methods used in this work is that they provide little information on the total fungal biomass produced as a function of time. In the future, such information in concert with the data on hyphal growth rates could be used to construct a more comprehensive picture of mycelial colonization rates of gypsum and other building materials. Data on biomass formation could be obtained from enhanced post‐processing of the movie data, or from other macroscopic methods designed specifically for the task, such as ergosterol determination (Nielsen *et al*., 2004) or the assessment of thermal output (Wadso, 1997).

Future work should include using the methods presented here to gain further insight in the effects of desiccation of hyphal growth. More complex *RH* schemes could be used to investigate the effect of multiple desiccations on subsequent development, to support the work of previous authors on the effects of cyclic humidity conditions (Adan, 1994; Viitanen and Ojanen, 2007; Johansson *et al*., 2013). On the other hand, as previously mentioned, a desiccation actually consists of two consecutive shocks, so investigating the effects of these shocks separately with an even simpler *RH* scheme might prove valuable for obtaining a more structured insight in the fungal response to desiccation. The methods could also be used to test novel compounds or materials that inhibit growth of moulds on indoor surfaces under simulated real conditions. Another interesting extension of the current work might be to investigate the response of other species, which might respond to transient humidity conditions differently. Indeed, there are indications that phylloplane indoor species of the *Cladosporium* genus (Park, 1982; Segers *et al*., unpublished data) whitstand transient humidities better than *Penicillium*.
